# 3VSR: Three Valued Secure Routing for Vehicular Ad Hoc Networks using Sensing Logic in Adversarial Environment

**DOI:** 10.3390/s18030856

**Published:** 2018-03-14

**Authors:** Muhammad Sohail, Liangmin Wang

**Affiliations:** School of Computer Science and Communication Engineering, Jiangsu University, Zhenjiang 212013, China; engrsohailaslam@gmail.com

**Keywords:** trust model, sensing logic, dirichlet distribution, AODV, Vehicular Ad-Hoc Networks

## Abstract

Today IoT integrate thousands of inter networks and sensing devices e.g., vehicular networks, which are considered to be challenging due to its high speed and network dynamics. The goal of future vehicular networks is to improve road safety, promote commercial or infotainment products and to reduce the traffic accidents. All these applications are based on the information exchange among nodes, so not only reliable data delivery but also the authenticity and credibility of the data itself are prerequisite. To cope with the aforementioned problem, trust management come up as promising candidate to conduct node’s transaction and interaction management, which requires distributed mobile nodes cooperation for achieving design goals. In this paper, we propose a trust-based routing protocol i.e., 3VSR (Three Valued Secure Routing), which extends the widely used AODV (Ad hoc On-demand Distance Vector) routing protocol and employs the idea of Sensing Logic-based trust model to enhance the security solution of VANET (Vehicular Ad-Hoc Network). The existing routing protocol are mostly based on key or signature-based schemes, which off course increases computation overhead. In our proposed 3VSR, trust among entities is updated frequently by means of opinion derived from sensing logic due to vehicles random topologies. In 3VSR the theoretical capabilities are based on Dirichlet distribution by considering prior and posterior uncertainty of the said event. Also by using trust recommendation message exchange, nodes are able to reduce computation and routing overhead. The simulated results shows that the proposed scheme is secure and practical.

## 1. Introduction

**Figure 1:**
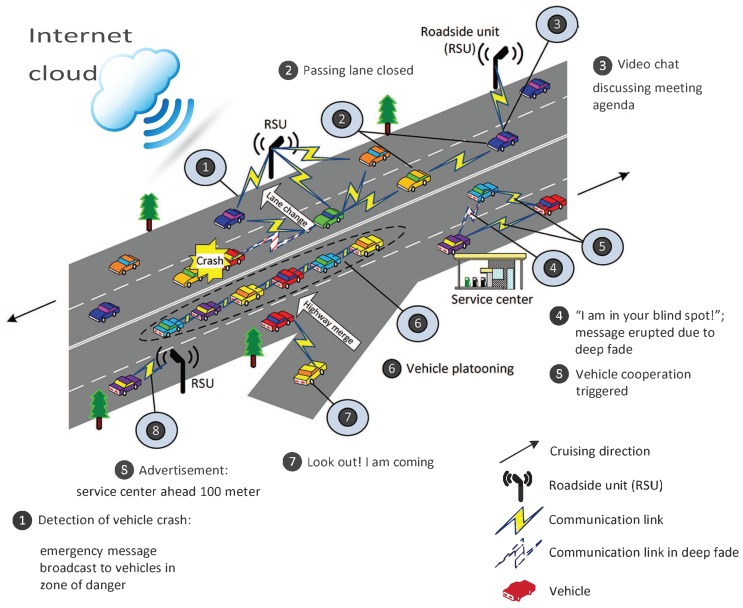
A possible future scenario in vehicular networks.

Recently, Vehicular ad hoc networks (VANets) are emerged as challenging and advanced networks, having lots of sensors and onboard devices for V-2-V (vehicle to vehicle), V-2-I (vehicle to infrastructure) and V-2-P (vehicle to pedestrian) communication, in addition with high speed and random topologies. As VANET is one of the important parts in implementing intelligent transportation systems (ITS) in which trust management [1,2,3] can play a vital role to improve traffic efficiency, human safety and reduce energy consumption. At the same time, with the increasing popularity of sensing technologies and hand held devices, a new sensing paradigm, mobile crowd sensing, attracts attention from both academia and industry [4]. This new sensing paradigm leverages the power of crowds for large scale sensing tasks and fuels the evolution of the Internet of Things (IoT). Today, vehicles on a highway can be a great opportunity for resource sharing, infotainment and commercial advertisement in an efficient manner. A possible future scenario of vehicular networks can be seen in Figure 1, these distributed networks will rely on each other for resource sharing, which demand secure communication to be disseminate. As VANET, due to its openness not only face security issues that described previously [5], but also come up with new challenges like high speed, large scale network that makes VANET a truly challenging network [6]. As a result, number of research efforts have been made to ensure the credibility of sensed data. [7]. Many authors tried to provide solutions using cryptographic and certificate exchange methods for securing ad hoc networks [8,9]. Similarly, most of the anonymous routing protocols like ANODR [10], AASR [11], also based on cryptographic and public key infrastructure mechanism for establishing secure connection, thus increases routing overhead. One possible solution is to establish the trust management system for evaluating the trustworthiness of volunteer contributions in participatory sensing applications [5].

Further, trust management succeeded to handle many security issues with lightweight solutions. Trust management enforcement in ad hoc networks enables system to derive collaboration, avoid untrusted and malicious nodes and improve network performance [12]. Jin et al. in [13] give a detail information about trust management in VANET and also highlight the challenges for its deployment. Kannan and parasat in [14] feasibly describe various trust computing approaches that are geared towards VANET. However, many trust management solutions rarely considered uncertainty as important notion in these ad hoc networks and that needs careful treatment. To manage uncertainty between distributed nodes author in [15] proposed subjective logic for determining probabilistic uncertain values over [0, 1]. This subjective logic succeeded to reduce computation overhead with simple network topology, while comes up as information loss by network canonization in complex and random network topology [16]. Precisely, to tackle with above situation liu et al. in [17,18] proposed Three Valued Subjective Logic to manage the uncertainty more significantly. Despite the existing methods, vehicular networks still need a comprehensive research and lightweight solutions.

In this paper, we propose a secure extended routing protocol, which is called 3VSR, to credit the user by establishing a trust and reputation system. In this system, a group of vehicles is able to authenticate each other and to distinguish the well-behaved and badly behaved vehicles according to their trust scores. To mitigate the negative impacts of those malicious user vehicle, we design an vehicle authentication algorithm for our proposed scheme to exclude their impact. The contributions of this paper can be summarized as follows:

- First, we take advantage of the unique features of VANET [19,20], e.g., high dynamics, hybrid architecture, and vehicle-to-infrastructure (V-2-I) and vehicle-to-vehicle (V-2-V) communications, to propose our 3VSR scheme. Specifically, the high dynamics ensure the real-time update of feedback. The hybrid architecture, i.e., vehicles, roadside units (RSUs), server, and trust authority (TA), enables the storage of feedback and the computation of trust scores.
- A secure routing is proposed using sensing logic as trust model to enhance security of VANET, as efficient multi-hop trust assessment technique. This trust model is capable of assessing trust between multi path, arbitrary and bridge topologies.
- In our 3VSR, nodes perform trusted routing behavior mainly according to the trust relationship between them, a node that will behave malicious eventually denied from the network. Also, system performance is improved by means of 3VSR as anonymous routing, that avoid verifying and requesting certificate exchange at each step. This also helps in minimizing computation and routing overhead.
- In this article, we have proposed sensing logic-based trust model, that despite highlighting posterior uncertainty during trust propagation also capable of correctly assess arbitrary topologies.

The rest of the paper sectioned as follows: In Section 2, we define the problem by formalizing the system model, adversary model and goals. In Section 3, we briefly highlight the AODV routing and Dirichlet distribution, which have been applied in trust model. In Section 4, we define the framework of the proposed scheme also with trust assumptions. In Section 5 trust model based on Sensing logic fundamentals are discussed in detail. In Section 6, trusted routing operation is revealed in detail. Experimental setup and results are explored in Section 7 and Section 8. Finally, related work is highlighted in Section 9, with conclusions in Section 10.

## 2. Problem Statement

Here our problem is defined by initiating the system model, adversary model, and design goal.

### 2.1. System Model

**Figure 2:**
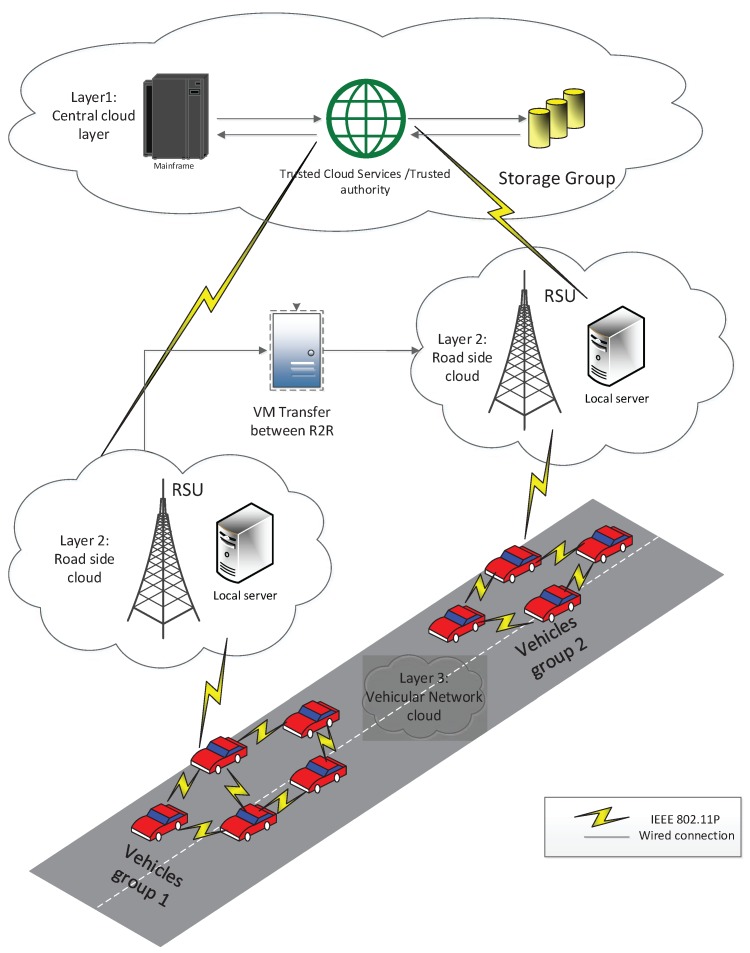
Three level layered Architectural network model for Vehicular ad hoc networks.

We make use of the advantage of the already existing architectural model for VANET [2] i.e., trusted authority, Server, RSUs and vehicles equipped with OBUs (On-Board Unit) and other sensing devices as shown in Figure 2. Due to high cost and commercial aspects of RSU here, we consider the limited number of RSUs. They can be installed in the main streets where many vehicles can pass and get register to the server using V-2-I communication. Further, our proposed system model can be implemented in future vehicular networks e.g., vehicular platoon.

*Central Cloud Layer CCL*: is a group of servers, trusted authority (TA) which have massive storage capacity and computational abilities. Central cloud can provides roadside services via V-2-I and V-2-R (vehicle to remote side unit) communication. CCL is responsible for registration of server, RSUs, and vehicles regarding brief behavioral history and profile also with digital certificate that is distributed soon after entering the coverage area.

*Remote Cloud layer RCL*: is a set of remote site units RSUs which gives general information and local services to vehicles on road. The main theme of our proposed system model is to enable vehicles to authenticate its neighbor without the central system to reduce extra overhead. After a complete round trip a vehicle’s group leader can update the correspondent information to the server via RSU.

*Vehicle Cloud Layer VCL*: is treated as local vehicles cloud on the road which can be built as a set of cooperating vehicles in different groups. Vehicles can share their resources via V-2-V communication using onboard modern sensors and computers. If the target vehicle has trust value above the minimum defined threshold then no need to verify its credibility using first and second-hand recommendation. If target vehicle has lower trust value i.e., below the threshold then the requesting vehicle can ask for certificate verification and also take first and second-hand recommendations from neighbor vehicles.

### 2.2. Adversary Model

The connected vehicles, on the road are generally more susceptible to various adversaries, and they can be compromised at any time after the VANET is formed. The adversary can be an outsider located in the wireless range of the vehicles, or the adversary can first compromise one or more vehicles and behave as an insider later. The malicious entity is able to jam, eavesdrop, forge, modify the communication between any vehicles in the range. The main goals of the adversary may include intercepting the normal data transmission, modifying or forging data, framing the benign devices by deliberately submitting fake recommendations, etc. Here, we proposed that only insider attacks may cause to change nodes behavior and act maliciously. The internal malicious users can randomly drop packets, as we have simulated this effect by considering half of the node malicious. Let, when there is impersonation attack by the fake routing packets, 3VSR can cope with this scenario by using authentication algorithm. In contrast standard AODV suffer more by means of packet loss and minimizing throughput due to lack of authentication in between neighbor nodes.

- Simple Attack (SA) An attacker may manipulate the compromised nodes not to follow normal network protocols and not to provide necessary services for other nodes, such as forwarding data packets or propagating route discovery requests. However, the compromised node will not provide any fake trust opinions when it is asked about other node’s trustworthiness.
- Black Hole Attack (BH) In this case malicious node doesn’t forward data packet, still remains active as using path initiated by other nodes to save its energy. In these attacks packets can be forged, altered and tend to behave as good nodes. Black Hole attacks are known as the basic byzantine attacks.
- Zigzag Attack (ZA): Sometimes sly attackers can alter their malicious behavior patterns so that it is even harder for the trust management scheme to detect them. For instance, they can conduct malicious behaviors for some time and then stop for a while (in that case the malicious behaviors are conducted in an on-and-off manner). In addition, the sly attackers can also exhibit different behaviors to different audiences, which can lead to inconsistent trust opinions to the same node among different audiences. Due to the insufficient evidence to accuse the adversary, it is generally more difficult to identify such sly attackers.

### 2.3. Design Goals

Following goals are some featured targets for the proposed scheme.

- Overhead: To reduce computational and routing overhead by using secure routing protocol and having limited number of RSUs.
- Resilience: How to tackle against adversary attack Modification attacks, Forgery attacks and Black Hole attacks during V-2-V authentication.
- Efficiency: The trust management scheme should be efficient in processing and having less time for convergence.
- Scalability and consistency: The proposed scheme should work fine even with high density of vehicles.

## 3. Preliminaries

To support mathematical operations on state space, we are interested in knowing the probability density function over multinomial opinion space. In case of binary opinion space it is well defined by Beta distribution but for multinomial scenario Dirichlet distribution is a solution [21].

### 3.1. Beta Distribution

Beta distribution is a continuous probability distribution defined over [0, 1] parametrized by two positive shape parameters, α and β [22]. These parameters appear as exponents of the random variable and control the shape of the distribution. The general expression for (PDF) of the beta distribution, for 0≤x≤1, having α,β>0 is a power function of the variable *x* and of its reflection (1−x) as follows:

(1) $$f\left(P_{b},P_{d}|\alpha ,\beta \right)=\frac{\Gamma (\alpha +\beta )}{\Gamma_{\left(\alpha \right)}+\Gamma_{\left(\beta \right)}}\times P_{b}^{\alpha -1}\times P_{d}^{\beta -1}$$

The probability density over binomial event spaces expressed as beta PDF’s and denoted as Beta (α,β). Also the probability expectation value of beta distribution is given by E(x)=α/(α+β). If *r* and *s* denotes the past observations of positive and negative behavior and *a* is a constant formed from an existing impression without solid evidences, e.g., prejudice, preference and general opinion obtained from hearsay., then we compute α,β as

α=r+2a,β=s+2(1−a)

Bijective mapping between opinion space and beta PDF parameters can be obtained using following equations.

(2) $$\left\{\begin{array}{c} b_{x}=\frac{r}{\left(r+s+2\right)}\Rightarrow r=\frac{2b_{x}}{ux}\\ d_{x}=\frac{n}{\left(r+s+2\right)}\Rightarrow s=\frac{2d_{x}}{ux}\\ u_{x}=\frac{2}{\left(r+s+2\right)}\Rightarrow u_{x}\neq 0 \end{array}\right.$$

### 3.2. Dirichlet Distribution

A Dirichlet distribution provides a solid mathematical foundation for measuring the ignorance of recommendation based on initial belief of an unknown event according to prior distribution. Compared with Beta distribution, which is more appropriate in a binary satisfaction level. Dirichlet distribution is more appropriate for multivalued satisfaction levels. In our case, the evaluation trustworthiness of user vehicles is described by continuous trust values. Therefore, we will use Dirichlet distribution to estimate opinion space of user vehicle recommended in the future and then build our trust model accordingly.

The Dirichlet distribution is a continuous sequence of observation having *k* possible outcomes with *k* positive real parameters $\alpha (x_{i}),i=1,\ldots ,k,$ in the form of compact vector notation $\vec{p}=p(x_{i}|1\leq i\leq k)$ denotes the *k*-component random probability variable and a vector $\vec{\alpha }=\left(\alpha_{i}|1\leq i\leq k\right)$ denotes random observation variable of *k* components such that ${\left[\alpha \left(x_{i}\right)\right]}_{i}^{k}$. The general form of Dirichlet distribution is as

(3) $$f\left(\vec{p}|\vec{\alpha }\right)=\frac{\Gamma \left(\sum_{i=1}^{k}\alpha \left(x_{i}\right)\right)}{\prod_{i=1}^{k}\Gamma \alpha \left(x_{i}\right)}\prod_{i=1}^{k}p{\left(x_{i}\right)}^{\alpha (x_{i})-1}$$

In sensing logic as evidences in opinion space have three condition (trust distrust, neutral), so modifying three valued evidence space to Dirichlet distribution as

(4) $$f\left(P_{b},P_{d},P_{n},P_{e}|\alpha ,\beta ,\gamma_{n},\gamma_{e}\right)=\frac{\Gamma (\alpha +\beta +\gamma_{n}+\gamma_{e})}{\Gamma_{\left(\alpha \right)}+\Gamma_{\left(\beta \right)}+\Gamma_{\left(\gamma_{n}\right)}+\Gamma_{\left(\gamma_{e}\right)}}\times P_{b}^{\alpha -1}\times P_{d}^{\beta -1}\times P_{n}^{\gamma -1}\times P_{e}^{\gamma -1}$$

where $\left(\alpha ,\beta ,\gamma_{n},\gamma_{e}\right)$ is the controlling vector and P(b,d,n,e) shows probability of belief, disbelief, posterior and prior uncertainty respectively. Let r,s and *o* denotes observed number of evidences that a node is trustworthy, untrustworthy or neutral. According to Dirichlet distribution, we have (α=r+1,β=s+1,γ=o+1)

Let us assume that the neighboring node had one prior evidence of each event (b,d,n). This assumption works satisfactorily because Dirichlet distribution can still works even when no event is observed i.e., (α=1,β=1,γ=1) and probability of each event will be 1/3. These three events are prior uncertainties, the four components in sensing logic opinion vector can be express as following after having Dirichlet distribution evidence spaces

(5) $$\begin{array}{c} b_{X}^{A}=\frac{r}{r+s+o+3},\;d_{X}^{A}=\frac{s}{r+s+o+3}\\ n_{X}^{A}=\frac{o}{r+s+o+3},\;e_{X}^{A}=\frac{3}{r+s+o+3} \end{array}$$

Here prior evidences is set to 3, its ratio to the number of observed event is $e_{X}^{A}$. it is worth noting that uncertainty *u* defined in subject logics is actually the prior evidence in sensing logic. Since in sensing logic expected probability of each event using above equation.

(6) $$\begin{array}{c} E\left(P_{b}\right)=\frac{\alpha }{\alpha +\beta +\gamma }=\frac{r+1}{r+s+o+3}=b_{X}^{A}+\frac{1}{3}e_{X}^{A}\\ E\left(P_{d}\right)=\frac{\beta }{\alpha +\beta +\gamma }=\frac{s+1}{r+s+o+3}=d_{X}^{A}+\frac{1}{3}e_{X}^{A}\\ E\left(P_{n}\right)=\frac{\gamma }{\alpha +\beta +\gamma }=\frac{o+1}{r+s+o+3}=n_{X}^{A}+\frac{1}{3}e_{X}^{A}\\ E\left(P_{e}\right)=\frac{3}{r+s+o+3}=e_{X}^{A} \end{array}$$

where the actual probability of each event is determined by baye’s rule using prior and posterior evidences.

### 3.3. Subjective Logic

To better understand sensing logic, we first briefly introduce the subjective logic. Subjective logic uses opinion-based probabilistic logic as input, output variables first proposed by A. Josang [16]. These opinion expresses uncertainties in probability values and identify the degree of ignorance in a particular subject such as trust. Let $\omega_{x}^{A}$ shows node A’s opinion about vehicle x trustworthiness in a specific context.

Evidence space in SL represented as ω=(b,d,u,a) where b,d,u and *a* show believe, disbelief and uncertainty over the range b,d,u∈[0,1] and b+d+u=1. The base rate a is a constant formed from an existing impression without solid evidences, e.g., prejudice, preference and general opinion obtained from hearsay. For example, if A always distrusts/trusts the persons from a certain group where X belongs to, then $a_{A}^{X}$ will be smaller/greater than 0.5. Based on the Beta distribution, the discounting and combining operation in subjective logic is as follows.

**Discounting operation**: Let A, B and C are three vehicles and $\omega_{B}^{A}=b_{B}^{A}+d_{B}^{A}+u_{B}^{A}$ shows A’s opinion about B trustworthiness and $\omega_{C}^{B}=b_{C}^{B}+d_{C}^{B}+u_{C}^{B}$ shows B’s opinion about C trustworthiness. Based on Beta distribution, $\omega_{C}^{A,B}=b_{C}^{A,B}+d_{C}^{A,B}+u_{C}^{A,B}$ shows A opinion about C using B’s advice to A.

(7) $$\left\{\begin{array}{c} b_{C}^{A,B}=b_{B}^{A}b_{C}^{B}\\ d_{C}^{A,B}=b_{B}^{A}d_{C}^{B}\\ u_{C}^{A,B}=d_{B}^{A}+u_{B}^{A}+b_{B}^{A}u_{C}^{B} \end{array}\right.$$

**Consensus operation**: Let, we have three vehicles A, B and C $\omega_{C}^{A}=b_{C}^{A}+d_{C}^{A}+u_{C}^{A}$ and $\omega_{C}^{B}=b_{C}^{B}+d_{C}^{B}+u_{C}^{B}$ be the opinions that vehicles A and B have about vehicle C’s trustworthiness such that by equations.

(8) $$\begin{array}{c} b_{C}^{A,B}=\frac{b_{B}^{A}u_{C}^{B}+b_{C}^{B}u_{C}^{A}}{u_{C}^{A}+u_{C}^{B}-u_{C}^{A}U_{C}^{B}},\;d_{C}^{A,B}=\frac{d_{C}^{A}u_{C}^{B}+d_{C}^{B}u_{C}^{A}}{u_{C}^{A}+u_{C}^{B}-u_{C}^{A}U_{C}^{B}},\;u_{C}^{A,B}=\frac{u_{C}^{A}u_{C}^{B}}{u_{C}^{A}+u_{C}^{B}-u_{C}^{A}u_{C}^{B}} \end{array}$$

Finally, the expected belief of an opinion $w_{B}^{A}$ is computed by $E\left(\omega_{B}^{A}\right)=b_{B}^{A}+a_{X}^{A}u_{B}^{A}$

### 3.4. Sensing Logic Fundamental

Opinion space in sensing logic is combined with these multiple values.

$$\omega_{B}^{A}=\left(b_{B}^{A},d_{B}^{A},n_{B}^{A},e_{B}^{A}\right)|a_{B}^{A}$$

$$b_{B}^{A}+d_{B}^{A}+n_{B}^{A}+e_{B}^{A}|a_{B}^{A}=1$$

where $b_{B}^{A},d_{B}^{A},n_{B}^{A},e_{B}^{A}|a_{B}^{A}$ represents belief, disbelief, posterior uncertainty during trust propagation and prior uncertain values. Where $a_{B}^{A}$ shows base rate, which is minimal probability value before the operation between A and B. It has the same definition in both SL and sensing logic, so we will ignore this notion unless it is necessary. The certainty of an opinion comes from $b_{B}^{A},d_{B}^{A}$, where $n_{B}^{A},e_{B}^{A}$ gives posterior and prior uncertainty values. For example if A has no interaction with B, then its opinion about trustworthiness of vehicle B is $\omega_{B}^{A}=\left(0,0,0,1\right)$. Later on, after some interaction with the neighbor vehicle B its opinion space can change like $\omega_{B}^{A}=\left(0.4,0.3,0.2,0.1\right)$ depending on successful and failed communication.

Further, unlike subjective logic, we define interpersonal trust as a trinary event (belief, distrust, neutral) instead of a binary event (belief, distrust), hence extend Beta distribution to Dirichlet distribution. Neutral state expresses the posteriori uncertainty generated by trust propagation, which is ignored in subjective logic. The introduction of neural state makes the operations in sensing logic different from subjective logic. Leveraging on this new definition, operations (discounting and combining) on trust are redesigned in sensing logic

### 3.5. Ad Hoc Routing Protocols

In ad hoc networks, we have mainly proactive and reactive routing protocols. Proactive routing requires high bandwidth space as, it maintains paths between source and destination even if they are not interacting. In AODV reactive routing paths are made on demand, so highly popular with ad hoc networks [23]. Trust model based on demand reactive routing is suitable for the distributed and pure ad hoc network [24].

#### Ad-Hoc on Demand Distance Vector Routing (AODV)

AODV is the most efficient reactive routing protocol for the ad hoc networks as paths are made on fly that is why called ad hoc on demand distance vector. On-demand is a key feature in AODV routing which means that paths are made on fly and maintained till they require each other services. In AODV each route request packet contains broadcast id, source sequence number, destination Ip address, hop counts and control flags. The sequence number identify the freshness of the routing packet and hop count contains the distance between the source node and the current node.

When source node S broadcast RREQ packet in search of destination node, each recipient of the RREQ packet looks up in its routing table. If receiving node doesn’t contain any information about the destination. It will create a backward path towards RREQ packet initiator and rebroadcast the routing request. Intermediate node receiving this RREQ and will generate a RREP message either if it has fresh route request information to satisfy or itself a destination. After that, this intermediate node will generate RREP packet and will forward it to the next hop towards RREQ initiator intermediary node, as indicated by source node routing table entry. when a node receives RREP packet, it update some fields in the routing table of RREP packet, and then forward it to the next hop and towards the originator (source node). After that a bidirectional path is setup and maintained as long as they required each other services [25].

Route maintenance is performed by either sending hello messages which acknowledged about the positive connectivity about the nodes and sender can listen these hello messages. Another way is to maintain local connectivity by some link or network layer intrusion detection mechanism [26]. Route maintenance can also be achieved using packet acknowledge in which nodes are in promiscuous mode and can overhear the packet transmission and easily detect malicious attacks [27].

## 4. Overview of Trusted AODV

### 4.1. Trusted Assumptions

Here, we made some assumptions for specific roles of entities, further we argue that we are mainly focuses on security solution to the routing behavior of network layer.

- Server: The server in central cloud layer is capable of having high storage capacity regarding brief history and profile also with vehicle Id and digital certificate that is distributed soon after entering the coverage area via RSU. Further, it is proposed that central server is under strong physical protection and not affected by adversaries.
- Remote Site Unit RSU: Here, we proposed that RSUs act as local trust manager for vehicles on road, but have limited storage capabilities as compared with server in CCL. Here RSU is used to manage the vehicle information e.g., IP address, public or private key etc for short time and update server after one complete trip via V-2-R communication.
- Vehicle: A vehicle can access all its neighbor vehicles and broadcast initial information about itself using V-2-V communication. A user vehicle after interacting provide their feedback about other vehicle. When a new vehicle join the group, the uncertainty towards it is normally high, so its trust value is evaluated after observing its behavior also taking advise from neighbor vehicles. At start, the new incoming vehicle also prove its credibility via exchange of digital certificate, which helps other vehicles to reduce uncertain opinion about it. Once the trust relationship establish vehicles can use our secure routing protocol to reduce extra communication overhead.

### 4.2. Framework for 3VSR

**Figure 3:**
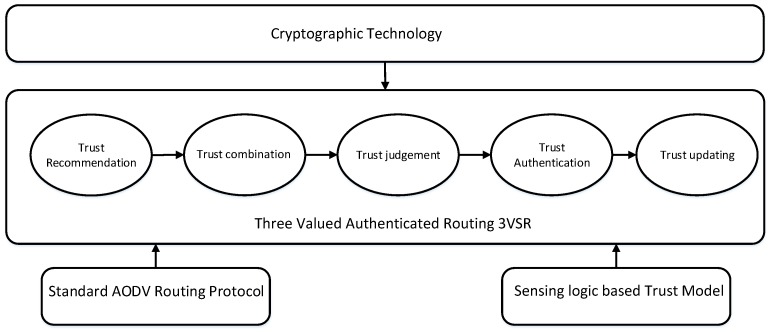
Frame work for the proposed scheme, the standard AODV is applied at second stage with enabling sensing logic-based trust model, while cryptographic technology is considered to take effect before this operation.

There are mainly three parts in the 3VSR framework i.e., standard AODV routing protocol, trust model and secure routing. Using our trust model, the 3VSR completes the procedure as trust recommendation, trust combination, trust judging, trusted routing behaviors, advance cryptographic routing behaviors and trust updating. From Figure 3, we can see that the relationship and structure between these entities. The general procedure for establishing trust relationships among nodes and for performing routing discovery is described as follows.

Let us consider the beginning stage when new nodes initiates communication with the network but they are uncertain about each other at beginning. In distributed VANET a node is free to move and join different network, their recent partners will evaluate their trust levels. In most previous work, the new comers are not aware of their forwarding behavior and thus set the trust level to null. This raises the alarm that the node will possibly be excluded from future routing. This approach is not feasible with a highly dynamic network. In our scheme, we did not considered the extreme values for new comers i.e., (honest, dishonest) till the network initialization. However, the uncertain opinion toward new joining node is set as u=(0,0,0,1), so at least it has a chance to prove its credibility by verifying digital certificate and minimize uncertainty.

After this initial activity, having some successful or failed, the communication node *A* can change its opinion about node *B*’s behavior using a trust update algorithm. After establishing bidirectional communicating path nodes can use our secure routing protocol for operation. As trust is asymmetric, mobile nodes uses second hand observation given by its neighbors, and finally combines into a single trust value. Notice that a node can join the existing VANET through many ways and several security algorithms can be used to run this operation. In this framework trust establishment and the route discovery are all treated by node’s cooperation without any third or central party.

## 5. Trust Model

We have used advanced sensing logic framework defined in preliminary section as our trust model. Following are the major definitions in sensing logic.

**Definition** **1.** “Trust representation”

*An opinion metric in sensing logic can be represented as T=[B,D,N,E], where (B,D,N,E)∈[0,1] and B+D+N+E=1 also B,D showing probability of belief and disbelief and N,E correspond posterior and prior uncertainty of said event. An opinion metric $T_{1}=\left[0.7,0.2,0,0.1\right]$ and $T_{2}=\left[0.4,0.5,0,0.1\right]$, shows high and low trust values respectively*.

**Definition** **2.** “Mapping”

*Let’s $T_{Y}^{X}=\left[B,D,N,E\right]$ be vehicle Y’s opinion about vehicle X’s trustworthiness in a VANET, and let r,s and o denote the observed piece of evidence that a vehicle is reliable, fake or neutral. Using Equation (5), given in preliminary section, we can map evidence space to opinion spaces*.

**Definition** **3.** “Trust Combination”

*In our trust model, A vehicle will make a relative judgment about the neighbor vehicle by means of first and second hand observation. First hand observation comes from direct or self experience and second hand observation comes as advise by other neighbors or friends. These two observation are combined through consensus and discounting operations, and a final opinion is computed towards target vehicle*.

**Consensus combination**: Let $S_{X}$, $S_{Y}$ and $S_{Z}$ be three vehicles. Then $T_{Y}^{X}=\left[B_{Y}^{X},D_{Y}^{X},N_{Y}^{X},E_{Y}^{X}\right]$ and $T_{Z}^{X}=\left[B_{Z}^{X},D_{Z}^{X},N_{Z}^{X},E_{Z}^{X}\right]$, shows opinions of vehicle $S_{Y}$ and $S_{Z}$ about truthfulness of vehicle $S_{X}$. Their consensus opinion space is defined as

(9) $$\begin{array}{c} B_{Y,Z}^{X}=\frac{E_{Z}^{X}B_{Y}^{X}+E_{Y}^{X}B_{Z}^{X}}{E_{Y}^{X}+E_{Z}^{X}-E_{Y}^{X}E_{Z}^{X}},\;\;D_{Y,Z}^{X}=\frac{E_{Z}^{X}D_{Y}^{X}+E_{Y}^{X}D_{Z}^{X}}{E_{Y}^{X}+E_{Z}^{X}-E_{Y}^{X}E_{Z}^{X}}\\ N_{Y,Z}^{X}=\frac{E_{Z}^{X}N_{Y}^{X}+E_{Y}^{X}N_{Z}^{X}}{E_{Y}^{X}+E_{Z}^{X}-E_{Y}^{X}E_{Z}^{X}},\;\;E_{Y,Z}^{X}=\frac{E_{Y}^{X}E_{Z}^{X}}{E_{Y}^{X}+E_{Z}^{X}-E_{Y}^{X}E_{Z}^{X}} \end{array}$$

The trust value using subjective opinions with consensus combining provide more flexible trust model of the real world. By referring to Equation (9), the consensus of trust opinions generated by vehicles ${si}_{i=1}^{n_{j}}$ in time interval *t* about vehicle $s_{j}$ is

$$T_{1}^{j,t}⊕\ldots ⊕T_{i}^{j,t}⊕\ldots .⊕T_{n_{j}}^{j,t}=T_{1,\ldots ,i,\ldots ,n_{j}}^{j,t}$$

**Discounting combination**: Let $S_{X}$, $S_{Y}$, and $S_{Z}$ be three vehicles. Then, we can present algebraically as $T_{X}^{Y}=\left[B_{X}^{Y},D_{X}^{Y},N_{X}^{Y},E_{X}^{Y}\right]$ and $T_{Y}^{Z}=\left[B_{Y}^{Z},D_{Y}^{Z},N_{Y}^{Z},E_{Y}^{Z}\right]$ shows opinions of $S_{X}$ about $S_{Y}$ trustworthiness and $S_{Y}$ about truthfulness of vehicle $S_{Z}$. Their discounting opinion is defined as $T_{X,Y}^{Z}=T_{X}^{Y}⊗T_{Y}^{Z}=\left[B_{X,Y}^{Z},D_{X,Y}^{Z},N_{X,Y}^{Z},E_{X,Y}^{Z}\right]$, shows $S_{X}$ opinion on vehicle $S_{Z}$ as advised by vehicle $S_{Y}$.

(10) $$\begin{array}{c} B_{X,Y}^{Z}=B_{X}^{Y}B_{Y}^{Z},\;D_{X,Y}^{Z}=B_{X}^{Y}D_{Y}^{Z}\\ N_{X,Y}^{Z}=1-B_{X,Y}^{Z}-D_{X,Y}^{Z}-E_{Y}^{Z},\;E_{X,Y}^{Z}=E_{Y}^{Z} \end{array}$$

The discounting operation is used along a recommendation path of multiple vehicles about the particular one.

**Definition** **4.** *Let $S_{X}$ and $S_{Y}$ be two vehicles. Then $\left[T_{Y}^{X,t_{1}},\ldots ,T_{Y}^{X,t_{n}}\right]$ shows opinions of vehicle $S_{Y}$ about trustworthiness of $S_{X}$ for time intervals $\left[t_{1},\ldots ,t_{n}\right]$ respectively, where $T_{Y}^{X,t_{n}}=\left[B_{Y}^{X,t_{n}},D_{Y}^{X,t_{n}},N_{Y}^{X,t_{n}},E_{Y}^{X,t_{n}}\right]$. Vehicle $S_{Y}$’s opinion on $S_{X}$ trustworthiness in different time intervals can be combined as $\left[t_{1},\cup \ldots \cup ,t_{n}\right]$ is defined as*

(11) $$\begin{array}{c} T_{Y_{certainty}}^{X,t_{1}\cup \ldots \cup t_{n}}=\left[B_{Y}^{X,t_{1}\cup \ldots \cup t_{n}},D_{Y}^{X,t_{1}\cup \ldots \cup t_{n}}\right],\;T_{Y_{Uncertainty}}^{X,t_{1}\cup \ldots \cup t_{n}}=\left[N_{Y}^{X,t_{1}\cup \ldots \cup t_{n}},E_{Y}^{X,t_{1}\cup \ldots \cup t_{n}}\right]\\ B^{X,t_{1}\cup \ldots \cup t_{n}=1/n\left(B_{Y}^{X,t_{1}}+\ldots +B_{Y}^{X,t_{n}}\right)},\;D^{X,t_{1}\cup \ldots \cup t_{n}=1/n\left(D_{Y}^{X,t_{1}}+\ldots +D_{Y}^{X,t_{n}}\right)}\\ N^{X,t_{1}\cup \ldots \cup t_{n}=1/n\left(N_{Y}^{X,t_{1}}+\ldots +N_{Y}^{X,t_{n}}\right)},\;E^{X,t_{1}\cup \ldots \cup t_{n}=1/n\left(E_{Y}^{X,t_{1}}+\ldots +E_{Y}^{X,t_{n}}\right)} \end{array}$$

The certainty of an event mainly comes from belief and disbelief, while uncertainty includes prior and posterior state of an event. Using Definition 3 and 4, we define trustworthiness $\gamma^{j}$ using entities consensus operation to combine trust opinions generated by vehicles ${\left\{S_{j,i}\right\}}_{i=1}^{n_{j}}$ in time interval ${\left\{t\right\}}_{t=t_{1}}^{t_{n}}$ as

(12) $$\gamma^{j}=T_{1,\ldots ,i,\ldots ,n_{j}}^{j,ti\cup \ldots \cup t_{n}}$$

The $\gamma^{j}$ can be calculated with respect to vehicle consensus or time interval method. The vehicle consensus is given as:

$$\gamma^{j}=T_{1,\ldots ,i,\ldots ,n_{j}}^{j,ti\cup \ldots \cup t_{n}}=T_{1}^{j,ti\cup \ldots \cup t_{n}}⊕\ldots ⊕T_{i}^{j,ti\cup \ldots \cup t_{n}}⊕\ldots ⊕T_{n_{j}}^{j,ti\cup \ldots \cup t_{n}}$$

and second with respect to time as follow

$$\gamma^{j}=T_{1,\ldots ,i,\ldots ,n_{j}}^{j,ti\cup \ldots \cup t_{n}}=B_{1,\ldots ,i,\ldots ,n_{j}}^{j,ti\cup \ldots \cup t_{n}},D_{1,\ldots ,i,\ldots ,n_{j}}^{j,ti\cup \ldots \cup t_{n}},N_{1,\ldots ,i,\ldots ,n_{j}}^{j,ti\cup \ldots \cup t_{n}},E_{1,\ldots ,i,\ldots ,n_{j}}^{j,ti\cup \ldots \cup t_{n}}$$

where

(13) $$\begin{array}{c} B_{1,\ldots ,i,\ldots ,n_{j}}^{j,ti\cup \ldots \cup t_{n}}=1/n\left(B_{1,\ldots ,i,\ldots ,n_{j}}^{j,t_{1}}+\ldots +B_{1,\ldots ,i,\ldots ,n_{j}}^{j,t_{n}}\right),\;D_{1,\ldots ,i,\ldots ,n_{j}}^{j,ti\cup \ldots \cup t_{n}}=1/n\left(D_{1,\ldots ,i,\ldots ,n_{j}}^{j,t_{1}}+\ldots +D_{1,\ldots ,i,\ldots ,n_{j}}^{j,t_{n}}\right)\\ N_{1,\ldots ,i,\ldots ,n_{j}}^{j,ti\cup \ldots \cup t_{n}}=1/n\left(N_{1,\ldots ,i,\ldots ,n_{j}}^{j,t_{1}}+\ldots +N_{1,\ldots ,i,\ldots ,n_{j}}^{j,t_{n}}\right),\;E_{1,\ldots ,i,\ldots ,n_{j}}^{j,ti\cup \ldots \cup t_{n}}=1/n\left(E_{1,\ldots ,i,\ldots ,n_{j}}^{j,t_{1}}+\ldots +E_{1,\ldots ,i,\ldots ,n_{j}}^{j,t_{n}}\right) \end{array}$$

According to Definition 3, each trust opinion has the same impact over time. Further, it is important that newer trust opinions have higher impact on trustworthiness, while previous trust opinions also taken into account. One solution is to use a time factor e.g., T∈[0,1] adding time impact into prior trust opinion, where greater *T* indicates newer opinion. More specifically, the time-aware trust opinion can be computed

(14) $$\begin{array}{c} T_{i-certainty}^{j,ti\cup \ldots \cup t_{n}}=B_{i}^{j,ti\cup \ldots \cup t_{n}},D_{i}^{j,ti\cup \ldots \cup t_{n}},\;\;T_{i-uncertainty}^{j,ti\cup \ldots \cup t_{n}}=N_{i}^{j,ti\cup \ldots \cup t_{n}},E_{i}^{j,ti\cup \ldots \cup t_{n}}\\ B_{i}^{j,ti\cup \;\ldots \cup t_{n}}=1/n\left(T^{n-1}B_{i}^{j,t_{1}}+\ldots +TB_{i}^{j,t_{n-1}}+B_{i}^{j,t_{n}}\right),\;D_{i}^{j,ti\cup \ldots \cup t_{n}}=1/n\left(T^{n-1}D_{i}^{j,t_{1}}+\ldots +TD_{i}^{j,t_{n-1}}+D_{i}^{j,t_{n}}\right)\\ E_{i}^{j,ti\cup \ldots \cup t_{n}}=1-B_{i}^{j,ti\cup \ldots \cup t_{n}}-D_{i}^{j,ti\cup \ldots \cup t_{n}},\;\;N_{i}^{j,ti\cup \ldots \cup t_{n}}=0 \end{array}$$

However, this extension is not considered here, and will be highlighted in future work. The reason is because assigning a suitable value of *T* is a challenging task and it needs careful investigation. For example T=0.99 and T=0.78, it is not clear, which one is more reasonable and how *T* varies over time. As the scope of this paper is secure routing using sensing logic, so we did not mention any results on time-aware solutions.

**Definition** **5.** “Original and Distorted opinion in sensing logic”

*Consider a discounting operation on two opinions as $△(w_{1},w_{2})$, we treat $w_{1}$ as distorting opinion $w_{2}$ as original opinion in trust propagation. Let, we have three vehicles A, B and C in series in which $w_{2}$ is actually direct opinion between vehicle B and C and $w_{1}$ is indirect (distorting) opinion between A and C*.

Since certain evidence from $w_{2}$ is distorted by $w_{1}$’s discounting operation and transferred to the posterior uncertainty of $w_{2}$ the evidence space of opinion $△(w_{1},w_{2})$ remains same as of $w_{2}$’s. So it is concluded that resulting opinion of a discounting operation shares exactly same evidence space as of original opinion. It is easy to prove that discounting operation is associative but not commutative.

$$△\left(w_{1},w_{2}\right)\neq △\left(w_{2},w_{1}\right)$$

$$△\left(△\left(w_{1},w_{2}\right),w_{3}\right)\equiv △(w_{1},△\left(w_{2},w_{3}\right)$$

$$△\left(△\left(△\left(w_{1},w_{2}\right),\ldots \right),w_{n}\right)\approx △\left(w_{1},w_{2},\ldots w_{n}\right)$$

Here, posteriori uncertainty is introduced using sensing logic to store neutral evidence eliminated from certainty space as trust propagates, while prior uncertainty is kept unchanged. Now consider these following equations.

**Lemma** **1.** $$△\left(w_{1},\Theta \left(w_{2},w_{3}\right)\right)\equiv \Theta \left(△\left(w_{1},w_{2}\right),△\left(w_{1},w_{3}\right)\right)$$

$$△\left(\Theta \left(w_{1},w_{2}\right),w_{3}\right)\neq \Theta \left(△\left(w_{1},w_{3}\right),△\left(w_{2},w_{3}\right)\right)$$

**Proof** **of** **Lemma** **1.** Proof of Lemma 1 is easy, so we omit the details here. Hence from the above discussion we concluded that in a “trust computation original opinions can be combined only once, while distorting opinion can be used number of times because they have minor effect on amount of evidence in resulted opinion”. ☐

## 6. Trusted Routing Operation in AODV

### 6.1. Node Model

**Figure 4:**
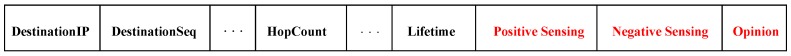
Extended routing table for AODV routing protocol having trust fields i.e., positive and negative sensing.

In our trust model, we have added trust field to the existing routing table i.e., positive and negative sensing as can be seen in Figure 4, which take place between the neighboring nodes and corresponding opinion metric is updated using Equation (5), with an increase or decrease of trust score.

### 6.2. Trust Judging Rules

For trust judgment, we have set the threshold 0.5, as can be seen in Table 1. This threshold value can be changed depending upon one’s system design and security level.

- If node *A* want to communicate with node *B* and if belief of *A* in *B* is ≥0.5, then *A* will trust *B* and start to route packet to node *B*.
- If disbelief opinion of *A* in node *B* i.e., >0.5, then *A* will not trust node *B* and will not route packets unless to verify it by certification or destroy it.
- If uncertainty of node *A* in node *B* is >0.5, then *A* will ask for digital signature for node *B* and waits for the verifying. If *A* successfully verifies *B’s* signature then *A* will start communication with *B* and reverse is also true.

### 6.3. Trust Updating Rule

When we talk about trust assessment then trust update is very important because it also counts good history of nodes and recommendation. We keep updating our repository due to dynamic nature of mobile ad hoc networks.

- If node *A* had successful communication with node *B* then its update the trust value by incrementing trust in that node. By successful communication we mean normal packet forward or RREP with in the time interval.
- If node *A* had failed communication with node *B* then it degrades trust values by decrementing the update counter.
- Every time field of successful or failed event is changed, opinion space values are recalculated using Equation (5).
- If node *B’s* routing entry is deleted from node *A* routing table due to expiry, then new opinion will set as $\omega_{B}^{A}=\left(0,0,0,1\right)$.

### 6.4. Trusted Information Exchange

**Figure 5:**
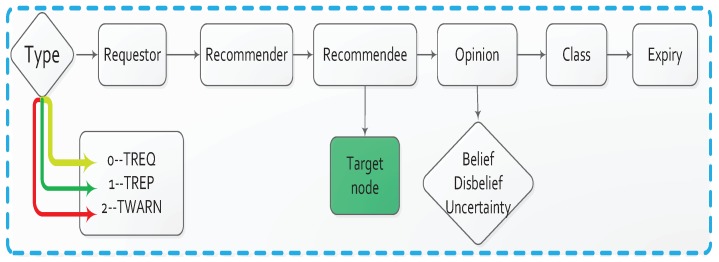
Trust information exchange between interacting nodes using three kind of route information i.e., TREQ, TREP and TWARN are shown with yellow green and red colors.

Existing trust models rarely consider exchange of trust information. However, trust information exchange is important in trust model applications and succeeded to reduce extra routing overhead. In our trust model, we derive an efficient trust information exchange mechanism by using three kind of messages that to be exchanged between neighbor nodes i.e., Trust Request Message (TREQ), TWARN (warning message) and Trust Reply Message (TREP) as shown in Figure 5. When a vehicle A wants to know neighbor vehicle B’s updated trust score, it will broadcast a TREQ message to its neighbors. This TREQ message follows the format given in Figure 5, with the Type field set to 0 and the trustee field filled with the IP address of vehicle B. In the same manner ,if destination node let say vehicle C receive this TREQ message, node C will reply with an TREP message. The Type field of this TREP is set to 1 and the opinion field is filled with the opinion values from C to B. Note that, in this recommendation protocol, a node can request or reply several opinion values of different vehicles simultaneously in one TREQ or TREP packet. In this way, we can efficiently exchange trust information without introducing much packets overhead.

In trusted routing discovery procedures, every routing request and reply carries trust information, including opinions towards originator vehicle A and destination vehicle B, which will be employed to calculate the credibility of A and B. When a vehicle is required to provide its certificate information, it will fill the fields of trust information with its own signature, as proposed by some traditional security solutions for mobile ad hoc networks.

A TWARN message, which is sent by a node to report invalid activity in original AODV procedure by type field set to 2, alarms other nodes to the worst trust warning. That is, If a vehicle A cannot verify certification then its opinion from the neighbor point of view set to be (0,1,0,0), which means total disbelief, and neighbor will broadcast an TWARN message and type field set to 2. Every vehicle before making any path to vehicle B first verify B’s trustworthiness then perform the corresponding update.

### 6.5. Trusted Routing Discovery

**Figure 6:**
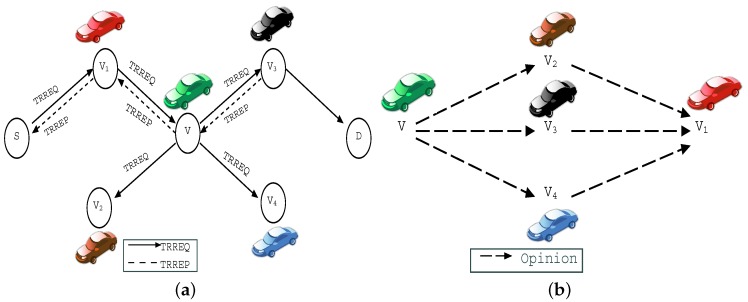
(**a**) General behavior of vehicles in performing trusted route discovery. (**b**) An example of trust recommendation from node *V* to node $V_{1}$.

In this section, we have described a general procedure for trust route discovery with an example shown in Figure 6a, also the route path from the source *S* to the destination *D* is uncovered. *S* will broadcast an TREQ message to discover a route path to *D*. Node *V* is an intermediate node along this path, and nodes $V_{1}$ to $V_{4}$ are its four neighbors. When *V* receives the re-broadcast TREQ message from $V_{1}$, it will perform such operations as illustrated in Algorithm 1.

Specifically, in the above algorithm, node V wants to verify node $V_{1}$’s trustworthiness. It then collects its neighbors recommendations towards $V_{1}$, and combines these opinions together using the combination operation as described in Section 5. Node *V* originally has opinions about $V_{1}$ as $w_{V_{1}}^{V}$. The indirect opinions it receives from its neighbors are: $w_{V_{1}}^{VV_{2}},w_{V_{1}}^{VV_{3}},w_{V_{1}}^{VV_{4}}$, where $w_{V_{1}}^{VV_{2}}$, shows opinion of *V* on $V_{1}$ as advised by node $V_{2}$ and so on. We can illustrate the trust recommendation relationships using Figure 6b, where the arrows denote opinion directions. First, $V_{1}$ calculates the following opinions using Discounting operation:

(15) $$\begin{array}{c} w_{V_{1}}^{VV_{2}}=△\left(w_{V_{2}}^{V},w_{V_{1}}^{V_{2}}\right)\\ w_{V_{1}}^{VV_{3}}=△\left(w_{V_{3}}^{V},w_{V_{1}}^{V_{3}}\right)\\ w_{V_{1}}^{VV_{4}}=△\left(w_{V_{4}}^{V},w_{V_{1}}^{V_{4}}\right) \end{array}$$

Second the new opinion can be combined as $w_{V_{1}}^{V}=w_{V_{1}}^{V(V_{2},V_{3},V_{4})}$, also by using Lemma 1

(16) $$\begin{array}{c} △\left(w_{V_{1}}^{V},\Theta \left(w_{V_{1}}^{V(V_{2},V_{3},V_{4})}\right)\right)\equiv \Theta \left(△\left(w_{V_{2}}^{V},w_{V_{1}}^{V_{2}}\right),△\left(w_{V_{3}}^{V},w_{V_{1}}^{V_{3}}\right)\right),\ldots \ldots ,△\left(w_{V_{4}}^{V},w_{V_{1}}^{V_{4}}\right)) \end{array}$$

After that newly computed opinion is judged according to our rules given in Algorithm 1.
**Algorithm 1** Trusted Routing Discovery.Receive an TREQ(S,D) or an TREP(S,D) from V1;/*Verify the trustworthiness of $V_{1}$*/Broadcast $TREQ(V_{1})$ to request the opinions from $V^{\prime }s$ neighbors to $V_{1}$;Receive opinions from $V^{\prime }s$ neighbors: $\omega_{V_{1}}^{V_{2}},\omega_{V_{1}}^{V_{3}},\omega_{V_{1}}^{V_{4}}$Combine these opinions together and get a latest: $\omega_{V_{1}}^{V}$Exchange opinions about $V_{1}$ with its neighbors $w_{V_{2},V_{3},V_{4}}^{V}$./* $\omega_{V_{1}}^{V}\neq U$ and Judge the next step using conditions set in Table 1 */***if***
$b_{V_{1}}^{V}\geq 0.5$trust $V_{1}$ and forward RREQ/RREP***elseif***
$d_{V_{1}}^{V}\geq 0.5$distrust $V_{1}$ for expiry time***elseif***
$u_{V_{1}}^{V}\geq 0.5$ request and verify digital certificate***else***/* the confidence about trustworthiness is decreased*/request and verify $V_{1}$ certificates, by default***endif***

### 6.6. Initiation of a Secure VANET

Let, we have a simple VANET having three vehicles $\left(V_{1},V,V_{3}\right)$ moving in a forward direction. $V_{1}$ has one neighbor and node *V* has two neighbor i.e., $V_{1}$ and $V_{3}$. At beginning each node has no entry in neighbor routing table, so the opinion metric is u=(0,0,0,1).

Now $V_{1}$ want to discover a route to node $V_{3}$ the process of node $V_{1},V$ and $V_{3}$ can be describe as

(1) $V_{1}$ broadcast RREQ requesting route path to node $V_{3}$ and waits for RREP in time *t* from *V*.
(2) *V* receive RREQ packet after that node *V* will check route to $V_{3}$ by checking opinion $\omega_{V_{1}}^{V}$ and $\omega_{V_{3}}^{V}$. As it is network initial stage so uncertainty will be high and currently no route to $V_{3}$.
  - Node *V* authenticates both the neighbors to verify certificate if $V_{1}$ passes, the successful event is increased by 1 and the new opinion $\omega_{V_{1}}^{V}=\left(0.33,0,0,0.67\right)$ is made. *V* also authenticates $V_{3}$ and revises the same process. If node $V_{1}$ fails the authentication, then new opinion will be $\omega_{V_{1}}^{V}=\left(0,0.33,0,0.67\right)$. *V* will not forward the packet till the expiry time.
  - If $V_{3}$ has also been authorized and *V*’s route table will be updated and *V* will re-broadcast the RREQ to $V_{3}$ after $V_{3}$ passed the authentication, *V* will forward the RREQ. If $V_{3}$ fails authentication process then opinion $\omega_{V_{3}}^{V}$ will be re-calculated accordingly.
(3) Node $V_{3}$ will also check $\omega_{V}^{V_{3}}$ and node *V*’s trustworthiness if *V* passes authentication, $V_{3}$ will generate an RREP packet to *V* and update its route table. If not, $V_{3}$ will drop the RREQ packet.

### 6.7. Trusted Route Maintenance

Route maintenance is analogous to trusted route discovery. Nodes uses trust information exchange rule to evaluate node trustworthiness and forward node authentication. So here extra detail about route maintenance algorithm is not mentioned.

## 7. Experimental Setup

We have performed a set of simulations using NS-2.35 [28,29] developed by Monarch research group. The simulations are conducted on a Lenovo G-580 machine with Intel Core-i3 processors of 2.66 GHz and 4-GB SDRAM running in a Ubuntu-16-Intel-64 bit operating system. NS-2 simulator has good support for simulating complete wireless network protocol model from physical and data link layer, Mac layer and routing layer to application layer. The basic parameters of our simulation are defined in Table 2.

### Evaluation Metrics

Following metrics has been evaluated to validate our proposed scheme.

- Packet Delivery Ratio (PDR): The ratio of the total number of data packets successfully delivered to the total number of data packets sent out by a source node.
- Packet Loss Ratio: It occurs when certain data packets traveling across a network fail to reach their destination. Packet loss is typically caused by network congestion. Packet loss is measured as a percentage of packets lost with respect to packets sent.
- Throughput: This value is calculated by dividing the overall number of messages received at destination node by the total messages sent from source nodes according to the following equation:
  $$\mathrm{Throughput}=\frac{\sum Total\;packets\;received\;}{\sum Total\;packets\;sent\;}$$
- Delay: It is very important factor to measure the efficiency of any communication system. Delay represents the time period that needs to route a packet from the source to the desired destination which depends on PDR value in the system and can be calculated using the following equation
  $$\mathrm{Delay}=\frac{Number\;of\;sending\;bits\;in\;the\;packet\;}{Throughput}$$
- Probability of Detection: It is the ratio between number of malicious nodes to the actual nodes present in the network. The malicious nodes are unable to make trust score up to threshold level, therefore 3VSR has high probability to detect these nodes.

## 8. Results and Discussions

- Test 1: Uncertainty analysis from initial stage to secure VANET.
- Test 2: Comparison between standard, AASR and our proposed AODV routing over different metric.
- Test 3: Comparison between Trusted vs standard AODV over different attack pattern.

### 8.1. Uncertainty Analysis from Initial Stage to Stable VANET

**Figure 7:**
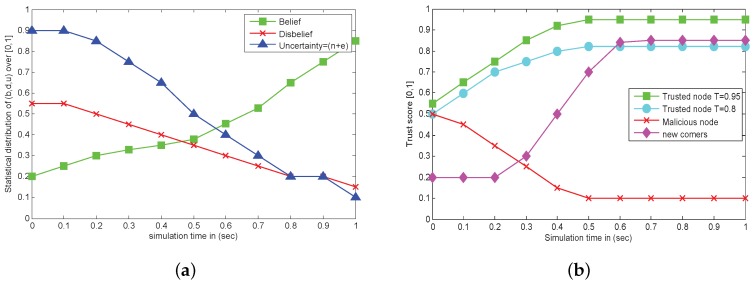
(**a**) Simulated analysis of belief, disbelief and uncertainty at network start. (**b**) Simulated behavior of trusted, malicious and new comers After some interaction.

In this scenario, we presented general behavior of vehicles at start of the network, as can be seen in Figure 7a. The uncertainty at the start is very high because the entities do not know much about each other, as $w_{B}^{A}=\left(0,0,0,1\right)$. After some interaction and gathering first and second hand observation, the opinion of entities are changes and uncertainty decreases. Keeping uncertainty high at start also help us to detect and tackle many malicious attacks, such as Modification, Forgery and Black Hole attacks.

In Figure 7b, a general behavior of nodes after some interaction is depicted. We can see that trusted nodes having values $t_{1}=0.95$ and $t_{2}=0.8$ are secure for communication. We can also see that once the user is attacked by an adversary they gradually decrease their trust score as they are unable to make good interaction with other entities. New incoming vehicle have less recommendation from neighbors at start, so its trust value increases with time after having some good actions.

### 8.2. Performance Comparison between Standard, AASR and Our Proposed 3VSR under Different Mobility and Attack Pattern

Here we have considered two different scenarios and observed the behavior of our proposed scheme with other protocol also.

#### 8.2.1. The Effect of Mobility Scenario

**Figure 8:**
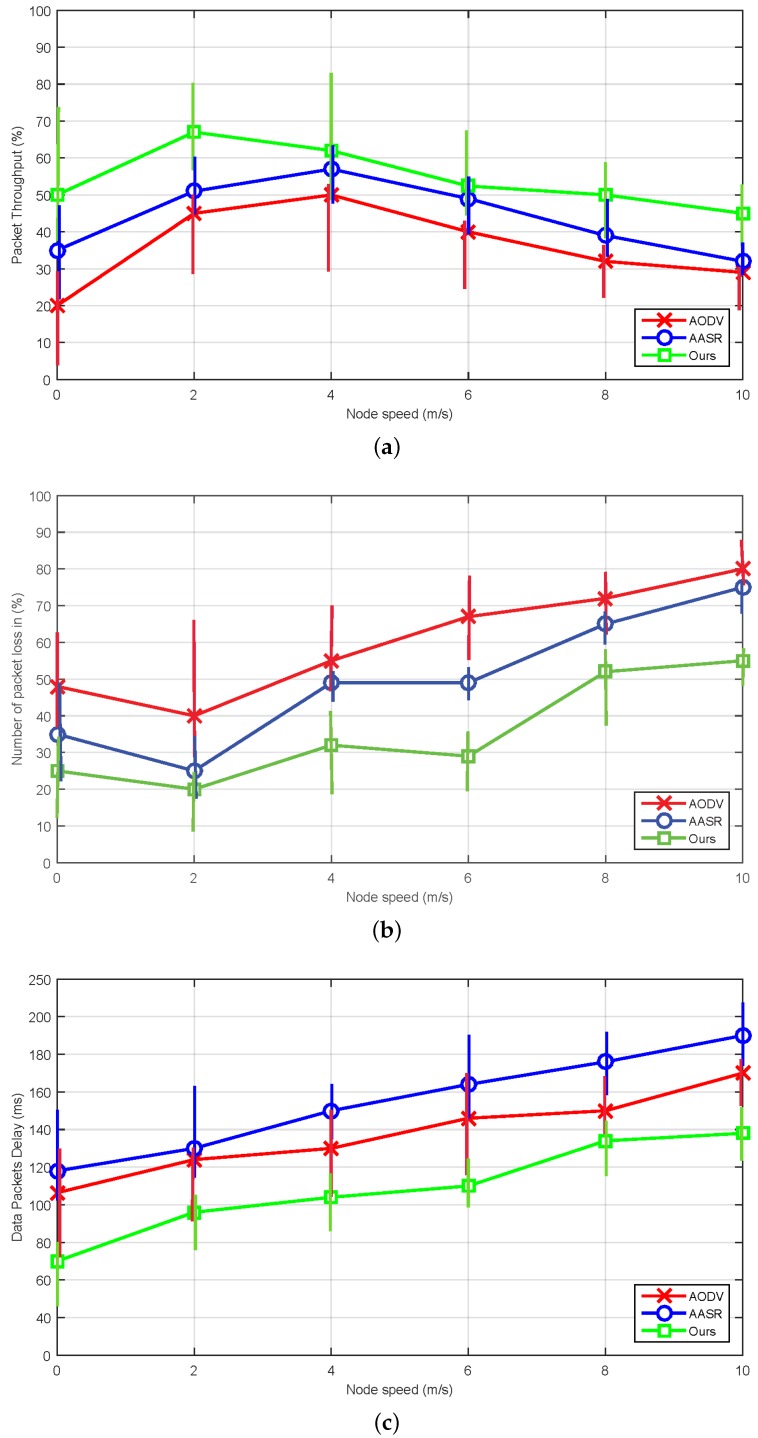
Performance comparison under different mobility setting: (**a**) Packet throughput. (**b**) Packet loss ratio. (**c**) End-to-end delay.

To simulate the adversarial effect, we have considered half of the nodes malicious i.e., 25 nodes having speed from 0 to 10 m/s in an arbitrary fashion. From Figure 8, we can see that 3VSR is better in throughput and packet loss ratio as compared with other routing protocols i.e., AODV and AASR. The performance of these protocols may be degraded under different mobility scenario. Despite the performance variation, our proposed scheme always achieves better throughput and lower packet loss as can be seen in Figure 8a,b, because it reduce computational overhead by avoiding complex cryptographic techniques. Meanwhile, AODV has lower delay values then AASR because if AASR is attacked by adversary it requires more cryptographic processing, which increases the delay. As a result, sometimes AASR performs worse than AODV, e.g., in the “slow” movement scenarios. The curves of the end-to-end delay are shown in Figure 8c, thus 3VSR is a better choice to adopt.

#### 8.2.2. The Effect of Malicious Attacks

**Figure 9:**
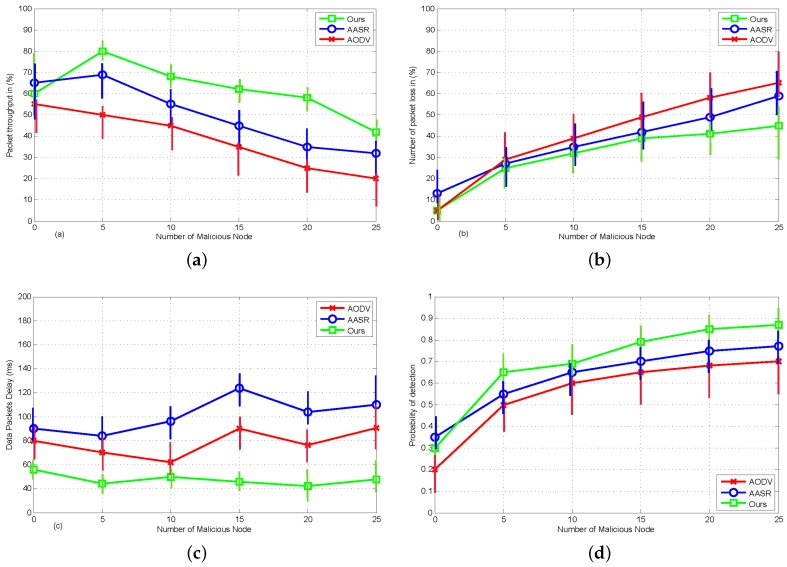
Performance comparison under different attack pattern: (**a**) Packet throughput. (**b**) Packet loss ratio. (**c**) End-to-end delay. (**d**) Probability of detection in between benevolent and malicious nodes.

Here, we have simulated the effect of malicious attacks i.e., half of the nodes are malicious under different mobility scenario i.e., node speed is 0 to 10 m/s. The results are plotted in Figure 9. From Figure 9a, we can see that the throughput of these protocol degraded with increase of malicious nodes. Since 3VSR has better ability to detect and tackle malicious attacks it outperform other routing protocols. Similarly, in Figure 9b our proposed scheme has less packet loss ratio than standard AODV and AASR. Since AODV is blind to the malicious attacks and takes no additional actions, its delay does not vary in the presence of different numbers of malicious nodes. Since AASR spends time in the route discovery after making security processing, their delay is higher than AODV, while 3VSR minimize this effect by using trust information exchange between neighbor nodes as can be seen in Figure 9c. Similarly, the probability of detection is higher in 3VSR as they denied the malicious users based on their threshold valued as can be seen in Figure 9d.

### 8.3. Performance Comparison between 3VSR vs. Standard AODV under Black Hole and Zigzag Attacks

**Figure 10:**
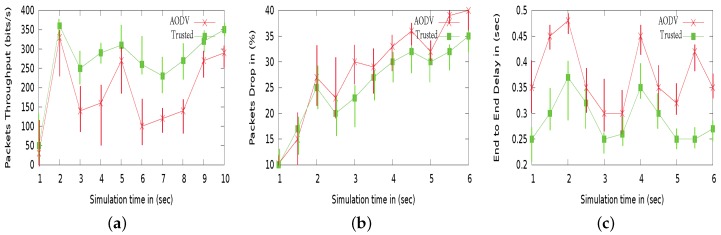
Effects of proportions of On-off attackers on the performances with parameter R = 250 m, V = 5 m/s, and N = 20: (**a**) Packet throughput (**b**) Packet drop ratio. (**c**) End-to-end delay.

In this simulation, we have made comparisons between trusted and malicious AODV under Black hole and On-off attack patterns. In Figure 10, we can see that secure routing has every aspect better than standard AODV. In Figure 10a, we can see the effect of an on-off attack, this scenario is also known as the changing behavior attack. As soon as nodes starts to behave badly with her neighbors their opinion metric changes in term of getting bad recommendation, thus lowering trust score as can be seen in Figure 10a. These on-off attacks are easily handled in our proposed secure routing, because intermediate nodes make forward node selection by calculating their trust score. Also in Figure 10b, we can see that the packet drop in secure routing is still much lower than standard AODV. The standard AODV do not care about malicious packets, so forward as gets from neighbor nodes. This factor though keeps minimizing the delay in standard AODV. Although in our scheme, routing overhead is reduced because of the established trust relationship between neighbor nodes, which helps in minimizing end to end delay, that can be seen in Figure 10c.

**Figure 11:**
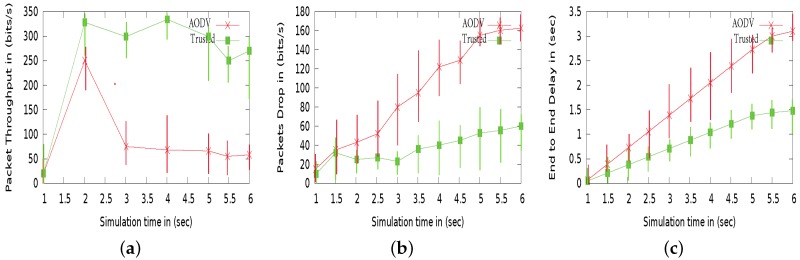
Comparison results between the original AODV and the trusted AODV under Black Hole attacks with parameters R = 250 m, V = 5 m/s, and N = 20: (**a**) packet Throughput. (**b**) Packet Drop in (%). (**c**) End-to-end delay.

The effect of a Black hole attack is strong, as nodes are completely compromised by altering or changing route packets and tend to behave well. In Figure 11a, we can see that nodes gets compromised by an adversary at start, so their packet throughput minimizes. Similarly, in Figure 11b, the number of packets dropped by secure and malicious AODV can be seen. The AODV routing without enabling trust model cannot tackle malicious attacks, nodes are compromised by these adversaries, which results in increase number of packets drop. Further, talking about end to end delay between secure and standard AODV. The delay factor increases due to high uncertainty and less interaction between malicious nodes over simulation time, while secure routing has capable of recognizing malicious nodes and ignoring interaction with them as can be seen in Figure 11c, thus able to minimize end to end delay.

## 9. Related Work

The related work can be divided into two parts, which are described as follows.

### 9.1. Misbehavior Detection for Ad hoc Networks

In recent years, many anonymous security solutions such as trapdoor, onion routing and group-based signature [30], and On-demand routing protocols [31], AASR [11] were proposed to detect the internal and external misbehavior actions by adversaries. These solutions heavily rely on cryptographic and signature-based mechanism to detect misbehavior activity. Another popular anonymous routing protocol i.e., ANODR also uses “ broadcasts with trapdoor information” for its design [10]. Although this scheme deals well with route anonymity and location privacy in MANET, although unsuitable for highly dynamic VANET as ANODR also relies on key exchange methods and lack of trust management and uncertainty concern between distributed nodes. Some authors proposed gathering trust information by setting nodes in promiscuous mode for neighbor nodes monitoring [27]. The possible drawback is depending on the success of the ability to access the content of packets in a header. Some researchers used intrusion detection system (IDS) in which there is an IDS probe on each node for monitoring purposes, which actually not an energy efficient solution for such distributed environment [26]. Many algorithms tend to give accurate trust assessment as trust is model with real number values [32,33], which totally ignore uncertainty between distributed nodes and the trust assessments are considered as inaccurate. The existing solutions rely heavily on the cryptographic mechanism and cause a huge delay in VANET. In modern vehicular networks e.g., intelligent transportation system (ITS) car maneuvers will rely more on disseminated information by neighbors, so building trust management can help in bringing lightweight solutions.

### 9.2. Trust Establishment and Management in Ad Hoc Networks

Trust management can help in building cooperation with unknown nodes to access the various observations and based on this make a reputation system to rank a good and badly behaved vehicle. The reputation system can be categorized to make sure credibility on which vehicle to cooperate with, and even to punish the untrusted vehicle. In our 3VSR, we have used two kind of sensing between neighbor vehicles, one is direct sensing that is comes from self experience with neighbor vehicle or through passive collection of some evidence by putting node into promiscuously mode or by packet acknowledgment in route discovery process. The second kind of sensing is indirect observation, it comes from one to many users as generally advise by fried or neighbor for a particular node. The indirect observation can be collected through surveys, monitoring past behaviors and recommendation of others. The main drawback of indirect observations are related to overhead, false report, trust distortion and collusion attacks [34].

Meanwhile, previously proposed secure routing protocols like CONFIDANT in [35] (Cooperation Of Nodes, Fairness In Dynamic Ad-Hoc Networks), to encourage the node cooperation and punish malicious nodes. A possible drawback of CONFIDANT is that an attacker may intentionally spread false alerts to other nodes that a node is misbehaving while it is actually a well-behaved node. Michiardi et al. [36] presented a solution called CORE to identify selfish nodes, and then compel them to cooperate in the following routing activities. Similar to CONFIDANT, CORE uses both a surveillance system and a reputation system to observe and evaluate node behaviors, but CORE only uses positive observation to be shared among nodes, this way, malicious nodes cannot spread fake charges to frame the well-behaved nodes. Patwardhan et al. [37] studied an approach in which the reputation of a node is determined by data validation. In this approach, a few nodes, which are named as Anchor nodes here, are assumed to be reliable, and thus the data they provide are regarded as trustworthy. In addition, there have been some other research efforts that aim to enhance the security, trust and privacy in ad hoc networks [38,39].

In addition, most of the existing trust management methods for ad hoc networks focus on assessing the trustworthiness of mobile nodes by collecting multiple evidences and analyzing behavioral history of the nodes. However, little attention has been paid to evaluate the trustworthiness of the data shared among these nodes and uncertainty management. Further, the lower uncertain values leads to high confidence in data shared between these distributed vehicles. Starting from probabilistic subjective logic, which comes up as promising technique to manage uncertainty between distributed nodes [40,41]. Xiaoqi et al. in [42] used subjective logic as advantage for forward node selection using trusted routing, which reduces extra computation and routing overhead. Some researchers also make use of fuzzy theory as logical reasoning [43]. These works contribute a lot to solve the uncertainty problem, although uncertainty generated as result of trust propagation rarely counted. Comparing to subjective logic, sensing logic distinguishes certain evidences that are distorted and transferred into the neutral state, referred as posterior uncertainty. Sensing logic also considers trust as distortion if it comes from one to many users as recommendation and separate them as original and distorted opinion by using Lemma 1, that already discussed in Section 5. Therefore, we rely on this new sensing logic for accurate trust assessment in vehicular ad hoc networks.

## 10. Conclusions

This manuscript proposed a three valued secure routing protocol between users vehicle in adversarial environment. The proposed scheme mainly focuses on establishing a trust model to improve the sensed data reliability and accuracy of the whole system. Our proposed trust model is capable of handing random network topologies and make accurate trust assessment by considering prior and posterior uncertainties between entities. Compared with standard and traditional routing protocols, 3VSR provides better throughput and lower packet loss ratio in different mobility scenarios. In our secure routing protocols using trust recommendation protocol, computational and routing overhead is reduced as vehicles after making trustful relationship, not really required certificate verification all the time. In addition, a silent feature of using a sensing logic-based trust model is to make the system design more flexible, the node threshold value can be set as per system requirements. In summary, the proposed trust mechanism with secure routing protocol using the sensing logic is able to reduce computation and extra routing overhead.

## Figures and Tables

**Table 1:** Trust judgment rules to authenticate a node at a certain security level.

$b_{X}^{A}$	$d_{X}^{A}$	$u(n_{X}^{A},e_{X}^{A})$	Action
		⩾0.5	Request to verify digital signature.
	>0.5		Distrust node till next request.
>0.5			Trust a node and share resources.
≤0.5	≤0.5	≤0.5	Request and verify authentication.

**Table 2:** Simulation parameters.

Examined Protocol	3VSR
Simulation time	100 (s)
Trust model	Sensing logic
Simulation area	1000 × 1000 (m)
Number of nodes	50
Transmission range	250 (m)
Propagation model	Two way ground reflection
Maximum speed	0–10 m/s
Physical link bandwidth	11 Mb
traffic type	CBR
Payload size	512 bytes
Packet rate	4 pkt/s
Routing Attacks	Black and Modification
Number of malicious nodes	0–25

## Abbreviations

The following abbreviations are used in this manuscript:

VANET	Vehicular Ad-Hoc Network
3VSR	Three Valued Secure Routing
AODV	Ad-Hoc On-Demand Distance Vector Routing
ITS	Intelligent Transportation System
RSU	Remote Side Unit
OBU	Onboard Unit
V-2-V	Vehicle to Vehicle communication
V-2-R	Vehicular to RSU
V-2-I	Vehicle to Infrastructure
CCL	Central Cloud Layer
RCL	Remote Cloud Layer
VCL	Vehicle Cloud Layer
